# Assessment of Greek Smokers’ Psychological Characteristics and Empathy While Smoking in Enclosed Public Spaces and Near Nonsmokers

**DOI:** 10.7759/cureus.22910

**Published:** 2022-03-07

**Authors:** Giorgos Iatrou, Konstantinos I Gourgoulianis, Evangelia Kotrotsiou, Mary Gouva

**Affiliations:** 1 Department of Respiratory Medicine, Faculty of Medicine, University of Thessaly, Larissa, GRC; 2 Department of Respiratory Medicine, Faculty of Medicine, General University Hospital of Larissa, Larissa, GRC; 3 General Department, University of Thessaly, Larissa, GRC; 4 Department of Nursing, University of Ioannina, Ioannina, GRC

**Keywords:** psychopathology, public areas, smoking behavior, empathy, psychosomatic symptoms, smoking

## Abstract

Background

Smoking presents a strong association between emotional intelligence and increased anxiety and depression. Empathy is a form of perception where people feel the emotional states of others as their own. The act of smoking expresses indifference to social norms and the health of nonsmokers, which speaks to smokers’ psychology. We conducted this study to identify the impact of smoking in psychology, empathy, and smoking behavior and examine the effect of smokers’ psychological characteristics and empathy toward smoking in enclosed public spaces and in front of nonsmokers.

Methodology

A primary, quantitative, synchronous, correlational, and nonexperimental research was accomplished using validated, reliable questionnaires. We used random sampling to acquire the study population consisting of 453 employees of public dining areas, owners of public dining areas, and medical and nonmedical students at the University of Larissa, Greece. Data were collected via self-completed questionnaires on participant demographic information and smoking habits. We used SPSS Statistics for Windows, version 24.0 (IBM Corp., Armonk, NY) to analyze the data with significance set at 5%. We also used independent samples t-test, Mann-Whitney U test, Spearman’s coefficient, chi-square test, and factorial analysis of variance with significance set at 5%.

Results

We found high levels of empathy in smokers with low psychosomatic symptoms. Smoking significantly affected levels of empathy (p<.001), annoyance when they are in a place where smoking is prohibited, someone else smoking (p<.001), recommendations of someone who smokes in a nonsmoking area to quit (p<.001), and hostility (p<.001). There was a statistically significant effect of double interaction sample category and smoking on empathy (p<.001). Smoking more than 15 cigarettes affected the levels of agreement in the perception that nonsmokers around them are bothered when they smoke (p=.004) and anxiety (p=.002). Perceptions about the annoyance of nonsmokers were negatively correlated with interpersonal sensitivity (p=.003), depression (p<.001), anxiety (p=.003), hostility (p<.001), paranoid ideation (p=.005), psychoticism (p=.001), and Global Severity Index (p=.006). Annoyance, when smoking is prohibited, was positively correlated with empathy (p=.001) while negatively correlated with somatization (p=.012) and hostility (p=.013). Smoking in prohibited places was related to somatization (p=.032), hostility (p<.001), and paranoid ideation (p=.001).

Conclusions

The purpose of this study was to examine the empathy and psychopathological characteristics of smokers in Greece. Smokers presented high levels of hostility and those who smoke more than 15 cigarettes per day indicated higher levels of anxiety than those who smoke less or not at all. Lower levels of empathy appeared in smokers, regardless of occupation. Smokers presented lower levels of annoyance when they are in a place where smoking is prohibited and someone else smokes. Participants with higher somatization, hostility, and lower empathy are less bothered when they are in a place where smoking is prohibited and someone else smokes. These findings could assist the development of communication materials aimed at smokers to help them understand that others nearby do not enjoy their smoking practices, especially in an enclosed area. These findings could also facilitate feasible antismoking laws with an overall goal to reduce smoking in a population.

## Introduction

Smoking is a significant health risk factor worldwide and is responsible for several serious diseases such as cancer, coronary heart disease, peripheral vascular diseases, chronic obstructive pulmonary disease, stroke, and peptic ulcers. Smoking during pregnancy carries severe consequences for the health of the fetus. Nicotine addiction is a neurobiological addiction and has been officially classified as a medical disease according to the Tenth Review of the Statistical Classification of Diseases and Related Health Problems [1,2].

Secondhand smoke (i.e., passive smoking) causes a significant disease burden with increased mortality rates. Passive smoking occurs in places where smokers and nonsmokers socialize. Smokers’ indifference to social norms and the health of nonsmokers is a critical component of their social and psychological health. Individuals expect to be evaluated negatively when they do not comply with social norms, and social and psychological aspects are critical to people’s behavior when smokers and nonsmokers are in the same place [3].

Studies for the control of the psychological parameters related to smoking previously focused on a smoker’s levels of anxiety and depression. However, a strong correlation exists between coercion, interpersonal sensitivity, depression, anxiety, anger, paranoid ideation, psychosis, and daily cigarette consumption. This correlation explains the psychological parameters contributing to smoking and its severity [4].

International research shows that Greece ranks the highest in smoking rates among Western European countries and Organisation for Economic Co-operation and Development countries [5]. Further, the mortality rates attributed to smoking-related factors are higher in Greece than in the other countries in the European Union [6]. Therefore, we conducted this study to identify the impact of smoking in psychology, empathy, and smoking behavior among smokers in Greece. In addition, we examined the effect of smokers’ psychological characteristics and empathy toward their smoking behavior in enclosed public spaces and in front of nonsmokers.

## Materials and methods

Research design

We conducted this quantitative, primary, synchronous, nonexperimental, and correlative study between and within-subjects at the University of Larissa, Greece, from 2016 to 2020. The study used validated questionnaires completed by study participants that collected demographic information and smoking habits.

Study population

We used random sampling to acquire the study population of 453 employees of public dining areas, owners of public dining areas, and medical and nonmedical students at the University of Larissa, Greece. Data were collected via self-completed questionnaires on participant demographic information and smoking habits. The public dining areas and employees were randomly selected.

Questionnaire

The questionnaire consisted of 120 questions and four sections. The first section collected demographic characteristics using seven closed-ended questions for gender, age, marital status, education, occupational status, monthly income, and sample category. The second section recorded smoking characteristics and behavior. Smoking characteristics were examined with eight closed-ended questions for smoking status (yes/no), the number of cigarettes smoked per day, smoking location (where they live, work, anywhere smoking is prohibited), and the presence of others in a nonsmoking area. Smoking behavior was examined with three Likert-type questions (1 = not at all, 2 = a little, 3 = moderately, 4 = much, 5 = very much) for the perceived levels of the annoyance of nonsmokers when they smoke, when they are in a place where smoking is prohibited, and when someone else smokes, and if they recommend someone who smokes in a nonsmoking area to quit.

The third section covered empathy as measured by the Toronto Empathy Questionnaire (TEQ) [7]. The TEQ includes 16 Likert-type questions (0 = never, 1 = seldom, 2 = sometimes, 3 = often, 4 = always) encompassing a wide range of behaviors related to the theoretical aspects of empathy. The TEQ presents good internal validity and high reliability in its review [7].

The fourth section recorded the psychosomatic symptoms. For this, we used the Symptom Check List 90-R (SCL90-R). The SCL90-R includes 90 Likert-type questions (0 = not at all, 1 = a little, 2 = moderately, 3 = much, 4 = very much) about psychopathology and provides an overview of a patient’s symptoms and their intensity at a specific point in time. The SCL90-R covers the following nine pathologies: somatization, obsessive-compulsive behavior, interpersonal sensitivity, depression, anxiety, hostility, phobic anxiety, paranoid ideation, and psychoticism [8].

Reliability

We conducted a reliability analysis (Table 1). Internal reliability was satisfying in all factors as the value of Cronbach’s alpha was more significant than 0.7 [9]. Using principal component analysis with varimax rotation and two components, all questions of the SCL90-R were placed in the first factor explaining 26.36% of the total variance. In contrast, the questions of TEQ were placed in the second factor explaining 7.33% of the total variance, results which indicate concept validity (Table 2) [10].

**Table 1:** Reliability analysis. TEQ: Toronto Empathy Questionnaire; SCL90-R: Symptom Check List 90-R; R: reverse item

Factors	Questions	Cronbach’s alpha
TEQ		
Empathy	1, 2R, 3, 4R, 5-6, 7R, 8-9, 10R, 11, 12R, 13, (14-15)R, 16	0.896
SCL90-R		
Somatization	1, 4, 12, 27, 40, 42, 48, 49, 52, 53, 56, 58	0.868
Obsessive-compulsive	3, 9, 10, 28, 38, 45, 46, 51, 55, 65	0.819
Interpersonal sensitivity	6, 21, 34, 36, 37, 41, 61, 69, 73	0.814
Depression	5, 14, 15, 20, 22, 26, 29, 30, 31, 32, 54, 71, 79	0.869
Anxiety	2, 17, 23, 33, 39, 57, 72, 78, 80, 86	0.866
Hostility	11, 24, 63, 67, 74, 81	0.823
Phobic anxiety	13, 25, 47, 50, 70, 75, 82	0.809
Paranoid ideation	8, 18, 43, 68, 76, 83	0.758
Psychoticism	7, 16, 35, 62, 77, 84, 85, 87, 88, 90	0.821
Global Severity Index	1–90	0.972

**Table 2:** Factor analysis using principal component analysis, varimax rotation, and two components. TEQ: Toronto Empathy Questionnaire; SOM: somatization; OBS: obsessive-compulsive; INT: interpersonal sensitivity; DEP: depression; ANX: anxiety; HOST: hostility; PHO: phobic anxiety; PAR: paranoid ideation; PSY: psychoticism

Questions	Component (KMO = 0.863)	
			1	2
SOM_12	0.706	
DEP_13	0.683	
ANX_3	0.667	
SOM_11	0.664	
DEP_8	0.658	
INT_6	0.657	
PAR_3	0.657	
ANX_6	0.657	
PSY_10	0.656	
ANX_2	0.654	
DEP_7	0.653	
INT_5	0.652	
PSY_5	0.651	
ANX_8	0.649	
ANX_9	0.649	
DEP_6	0.649	
ANX_1	0.645	
PSY_7	0.626	
SOM_3	0.625	
DEP_12	0.622	
PAR_5	0.621	
DEP_9	0.620	
HOST_4	0.611	
OBS_4	0.606	
OBS_8	0.603	
DEP_3	0.597	
ANX_5	0.595	
DEP_5	0.591	
OBS_9	0.590	
OBS_1	0.588	
ANX_4	0.586	
PHO_4	0.585	
INT_2	0.578	
PHO_1	0.569	
PSY_6	0.567	
OBS_6	0.566	
PHO_6	0.563	
INT_4	0.561	
SOM_8	0.560	
SOM_9	0.559	
ANX_7	0.557	
PSY_8	0.556	
DEP_10	0.556	
PSY_4	0.555	
PSY_2	0.555	
OBS_2	0.554	
PHO_5	0.552	
INT_8	0.538	
SOM_2	0.538	
HOST_5	0.529	
PSY_1	0.527	
PSY_9	0.527	
OBS_5	0.522	
ANX_10	0.520	
PHO_7	0.517	
HOST_1	0.510	
INT_7	0.509	
PAR_4	0.509	
SOM_6	0.507	
DEP_11	0.507	
SOM_5	0.503	
INT_3	0.503	
DEP_1	0.499	
PHO_2	0.492	
PHO_3	0.488	
HOST_6	0.488	
SOM_7	0.488	
SOM_10	0.476	
HOST_2	0.473	
PAR_2	0.472	
DEP_2	0.469	
OBS_7	0.466	
PAR_6	0.461	
PAR_1	0.458	
SOM_4	0.451	
INT_1	0.450	
OBS_3	0.441	
HOST_3	0.440	
DEP_4	0.439	
SOM_1	0.426	
OBS_10	0.406	
INT_9	0.400	
PSY_3	0.283	
TEQ_6		0.739
TEQ_16		0.737
TEQ_5		0.737
TEQ_14		-0.667
TEQ_12		-0.658
TEQ_11		0.646
TEQ_3		0.645
TEQ_13		0.611
TEQ_8		0.595
TEQ_1		0.563
TEQ_7		-0.562
TEQ_9		0.554
TEQ_15		-0.546
TEQ_4		-0.488
TEQ_2		-0.467
TEQ_10		-0.458
Variance (%)	26.36%	7.33%

Ethics

The University of Larissa, Department of Medicine Ethics Committee (17/5/2021) approved the study. The study was conducted following the Helsinki declaration for ethical principles for medical research involving human subjects. All participants were informed of the purpose of the investigation, assured of the confidentiality of all personal data, and gave their written consent. The questionnaires were completed by the participants in the researcher’s presence and with his help when necessary [11].

Statistical analysis

We used SPSS Statistics for Windows, version 24.0 (IBM Corp., Armonk, NY, USA) to analyze the data. Percentages and frequencies were used for categorical variables, while mean, standard deviation, and range were used for scale variables. Significance was set at 5%. The Shapiro-Wilk test was used to check the normality of variables. Independent samples t-test was used to compare means between two large samples (n≥30) or samples that are typically distributed, otherwise, the nonparametric Mann-Whitney U test was used. The Spearman coefficient was used to examine the correlation between non-normal-scale and ordinal variables. Analysis of variance 4 × 2 was used to examine the interaction of the sample category and smoking to empathy. We used the chi-square test to examine dependencies between categorical variables [12].

## Results

Demographic data

In total, 453 people participated in the study. Table 3 presents the demographic characteristics of the study population. There were slightly more male respondents than female respondents (233 males, 51.43%; 220 females, 28.75%). Most participants were aged 18 to 30 years (n=228, 70.2%), unmarried (n=357, 78.81%), with a bachelor’s degree (n=312, 68.87%), and working full or part time (n=249, 55.21%) with a monthly income of up to 1,000 euros (n=362, 95%). The population consisted of employees (n=140, 30%), medical students (n=129, 28.48%), owners of dining areas (n=116, 25.61%), and nonmedical students (n=68, 15.01%).

**Table 3:** Demographic characteristics of participants.

Demographics	Options	Ν (%)
Gender	Male	233 (51.43%)
	Female	220 (48.57%)
Age (in years)	18–25	203 (44.8%)
	26–30	115 (25.4%)
	31–40	109 (24.1%)
	41–66	26 (5.7%)
Marital status	Single	357 (78.81%)
	Married with children	77 (17.00%)
	Married without children	19 (4.19%)
Education	High school	50 (11.04%)
	Vocational training Institute	57 (12.58%)
	Bachelor	312 (68.87%)
	Master	34 (7.51%)
Occupational status	Unemployed	148 (32.82%)
	Occasionally	54 (11.97%)
	Part time	59 (13.08%)
	Full time	190 (42.13%)
Monthly income (in Euros)	0–500	185 (48.6%)
	501–1,000	177 (46.5%)
	>1,000	19 (5.0%)
Sample category	Employees	140 (30.91%)
	Medical students	129 (28.48%)
	Owners	116 (25.61%)
	Students of other studies	68 (15.01%)

Table 4 presents the smoking practices of the population. Most survey respondents were nonsmokers (n=288, 63.58%); only 165 respondents indicated they smoke (36.42%). Most smokers reported smoking 6-10 cigarettes per day (n=30, 43.48%) or 11-15 cigarettes per day (n=20, 28.99%). Over half of the respondents who smoke reported smoking where they live (58.18%), work (64.24%), and where smoking is prohibited by law (59.39%). Fewer than half indicated they smoke in front of friends (42.42%).

**Table 4:** Smoking characteristics.

Characteristic	Options	Ν (%)
Smokers	No	288 (63.58%)
	Yes	165 (36.42%)
Number of cigarettes you smoke per day	1–5	29 (33.72%)
	6–10	22 (25.58%)
	11–15	15 (17.44%)
	>16	20 (23.26%)
How many cigarettes do you smoke	1–5	10 (14.49%)
	6–10	30 (43.48%)
	11–15	20 (28.99%)
	>16	9 (13.04%)
Do you smoke in the place where you live?	No	69 (41.82%)
	Yes	96 (58.18%)
Do you smoke in your workplace?	No	59 (35.76%)
	Yes	106 (64.24%)
Do you smoke in places where smoking is prohibited under the law?	Νο	67 (40.61%)
	Yes	98 (59.39%)
When you smoke in a nonsmoking area who else is usually present?	Nobody	21 (12.73%)
	Children	7 (4.24%)
	Family	5 (3.03%)
	Friends	70 (42.42%)
	Unknown	62 (37.58%)

Descriptive statistics

Table 5 presents the descriptive statistics of smoking behaviors. Participants reported that they believe nonsmokers near them are moderately bothered when they smoke (mean=3.22, SD=0.99). Participant smokers were moderately bothered when they were in a place where smoking is prohibited, and when someone else was smoking (mean=3.00, SD=1.37). However, they reported that they seldom request an active smoker to stop smoking in an area where smoking is prohibited (mean=1.76, SD=0.79). As shown in Table 6, smokers showed a high level of empathy (mean=2.69, SD=0.65) but low levels of phobic anxiety, psychoticism, anxiety, somatization, interpersonal sensitivity, and depression.

**Table 5:** Descriptive statistics of smoking behaviors. SD: standard deviation

Question	Μean	SD	Range
How much do you think nonsmokers who are around you are bothered when you smoke?	3.22	0.99	1–5
How much does it bother you when you are in a place where smoking is prohibited and someone else smokes?	3.00	1.37	1–5
Do you recommend someone who smokes in a nonsmoking area to quit?	1.76	0.79	1–5

**Table 6:** Descriptive statistics of factors. SD: standard deviation

Factor	Μean	SD	Range
Empathy	2.69	0.65	0–4
Somatization	0.73	0.64	0–4
Obsessive-compulsive	1.00	0.65	0–4
Interpersonal sensitivity	0.85	0.64	0–4
Depression	0.86	0.69	0–4
Anxiety	0.72	0.66	0–4
Hostility	0.90	0.79	0–4
Phobic anxiety	0.50	0.61	0–4
Paranoid ideation	0.94	0.72	0–4
Psychoticism	0.62	0.57	0–4
Global Severity Index	0.79	0.55	0–4

First Research Question

Nonsmokers presented high levels of empathy (t=7.685, p<.001) and annoyance when they were in a place where smoking is prohibited, and when someone else smoked (t=10.99, p<.001). They also had high levels of requesting someone cease smoking in a nonsmoking area (t=5.70, p<.001) and low levels of hostility (t=-3.686, p<.001; Tables 7-9).

**Table 7:** Independent samples t-test between no smokers and smokers in psychological, empathy factors, and smoking behaviors. df: degrees of freedom; SD: standard deviation

Factor	Nonsmokers, mean (SD)	Smokers, mean (SD)	t-test	df	P-value
Empathy	2.87 (0.47)	2.36 (0.77)	7.685	235.26	.001
Annoyance when smoking is prohibited	3.47 (1.28)	2.19 (1.14)	10.99	374.89	.001
Recommend to quit smoking	1.91 (0.80)	1.50 (0.70)	5.70	451	.001
Somatization	0.69 (0.61)	0.79 (0.67)	-1.578	316.05	0.116
Obsessive-compulsive	1.01 (0.67)	0.96 (0.61)	0.804	451	0.422
Interpersonal sensitivity	0.88 (0.64)	0.78 (0.64)	1.553	451	0.121
Depression	0.85 (0.68)	0.87 (0.72)	-0.384	451	0.702
Anxiety	0.72 (0.68)	0.71 (0.61)	0.083	451	0.934
Hostility	0.79 (0.68)	1.09 (0.91)	-3.686	270.20	.001
Phobic anxiety	0.51 (0.62)	0.48 (0.60)	0.401	451	0.688
Paranoid ideation	0.92 (0.71)	0.99 (0.74)	-0.993	329.66	0.322
Psychoticism	0.60 (0.58)	0.66 (0.55)	1.034	451	0.301
Global Severity Index	0.78 (0.56)	0.81 (0.54)	0.521	348.46	0.603

**Table 8:** ANOVA 4 (employees, medical students, owners, and students of other studies) × 2 (no smokers, smokers) for empathy. ANOVA: analysis of variance; MS: mean square; df: degrees of freedom

Variable	df	MS	Frequency	P-value	η^2^
Sample category	3	3.290	10.525	.001	.066
Smoking	1	32.436	103.752	.001	.189
Sample category* smoking	3	3.878	12.404	.001	.077
Error	445	0.313			
Total	453				

**Table 9:** Mean value and 95% confidence intervals for empathy in sample categories for nonsmokers and smokers.

Category	Smoking	Μean	95% lower	95% upper
Employees	No	2.91	2.78	3.04
	Yes	2.71	2.57	2.85
Medical students	No	2.91	2.80	3.01
	Yes	2.38	2.14	2.61
Owners	No	2.66	2.53	2.78
	Yes	2.18	2.01	2.35
Students of other studies	No	3.15	2.95	3.34
	Yes	1.94	1.75	2.12

Age, marital status, and income level were not significantly associated with smoking (Tables 10-13). However, we saw a significant association between smoking status and gender, education level, occupational status, and type of respondent (Tables 14-16). We found that males who finished high school with vocational training were more likely to report smoking, while unemployed respondents and medical students were less likely to smoke.

**Table 10:** Age and smoking chi-square test.

			Age (in years)			
Chi-square (3)=5.244, p=0.155			18–25	26–30	31–40	41–66
Smoking	No	N	138	64	68	18
		%	47.9%	22.2%	23.6%	6.3%
	Yes	N	65	51	41	8
		%	39.4%	30.9%	24.8%	4.8%
	Total	N	203	115	109	26
		%	44.8%	25.4%	24.1%	5.7%

**Table 11:** Marital status and smoking chi-square test.

			Marital status		
Chi-square (2)=1.883, p=0.390			Single	Married with children	Married without children
Smoking	No	N	134	54	11
		%	81.2%	18.8%	3.8%
	Yes	N	223	23	8
		%	77.4%	13.9%	4.8%
	Total	N	357	77	19
		%	78.8%	17.0%	4.2%

**Table 12:** Education and smoking chi-square test.

			Education			
Chi-square (3)=22.229, p<.001			High school	Vocational training institute	Bachelor	Master
Smoking	No	N	20	29	212	27
		%	6.9%	10.1%	73.6%	9.4%
	Yes	N	30	28	100	7
		%	18.2%	17.0%	60.6%	4.2%
	Total	N	50	57	312	34
		%	11.0%	12.6%	68.9%	7.5%

**Table 13:** Monthly income and smoking chi-square test.

			Monthly income (in Euros)		
Chi-square (2)=0.977, p=0.614			0–500	501–1,000	>1,000
Smoking	No	N	106	110	12
		%	46.5%	48.2%	5.3%
	Yes	N	79	67	7
		%	51.6%	43.8%	4,6%
	Total	N	185	177	19
		%	48.6%	46.5%	5.0%

**Table 14:** Gender and smoking chi-square test.

			Gender	
Chi-square (1)=9.932, p=.002			Male	Female
Smoking	No	N	132	156
		%	45.8%	54.2%
	Yes	N	101	64
		%	61.2%	38.8%
	Total	N	233	220
		%	51.4%	48.6%

**Table 15:** Occupational status and smoking chi-square test.

			Occupational status			
Chi-square (3)=17.995, p< .001			Unemployed	Occasionally	Part time	Full time
Smoking	No	N	106	22	42	118
		%	36.8%	7.6%	14.6%	41.0%
	Yes	N	42	32	17	72
		%	25.8%	19.6%	10.4%	44.2%
	Total	N	148	54	59	190
		%	32.8%	12.0%	13.1%	42.1%

**Table 16:** Sample category and smoking chi-square test.

			Sample category			
Chi-square (3)=34.965, p< .001			Employees	Medical students	Owners	Students of other studies
Smoking	No	N	75	107	74	32
		%	26.0%	37.2%	25.7%	11.1%
	Yes	N	65	22	42	36
		%	39.4%	13.3%	25.5%	21.8%
	Total	N	140	129	116	68
		%	30.9%	28.5%	25.6%	15.0%

Participants who smoked more than 15 cigarettes per day were less likely to think (U=389.5, p=.004) that nonsmokers nearby are bothered when they smoke (mean rank >15 =29.98, mean rank 1-15 =47.60) than those who smoked fewer than 15 cigarettes per day. Participants who smoked more than 15 cigarettes per day also had higher levels of anxiety (U=353, p=.002) than those who smoked fewer than 15 cigarettes per day (mean rank >15 =58.85, mean rank 1-15 =38.85; Table 17).

**Table 17:** Mann-Whitney U test and independent samples t-test for psychological, empathy factors, and smoking behaviors regarding the number of cigarettes smoked.

Factor	1–15 cigarettes smoked (N=66)	>15 cigarettes smoked (N=20)	Statistic	P-value
Empathy	41.89	48.83	U=553.5	0.275
Annoyance of nonsmokers	47.60	29.98	U=389.5	.004
Annoyance when smoking is prohibited	45.91	35.55	U=501	0.087
Recommend to quit smoking	44.66	39.68	U=583.5	0.354
Somatization	42.14	47.98	U=570.5	0.358
Obsessive-compulsive	43.89	42.20	U=634	0.789
Interpersonal sensitivity	44.55	40.05	U=591	0.476
Depression	41.07	51.53	U=499.5	0.099
Anxiety	38.85	58.85	U=353	.002
Hostility	1.15 (1.05)	1.49 (0.71)	t (46.89)=-1.67	0.102
Phobic anxiety	43.76	42.65	U=643	0.859
Paranoid ideation	40.89	52.13	U=487.5	0.074
Psychoticism	42.42	47.05	U=589	0.465
Global Severity Index	41.70	49.45	U=541	0.223

Second Research Question

Smokers’ annoyance of nonsmokers was significantly negatively correlated with depression (p<.001) and hostility (p<.001). Smokers’ annoyance when smoking is prohibited was positively correlated with empathy (p=.001; Table 18). Participants who do not smoke in prohibited places have significantly lower levels of hostility (p<.001) and paranoid ideation (p=.001) than those who smoke where it is prohibited (Table 19).

**Table 18:** Spearman correlations between psychology and empathy factors with smoking behaviors.

Factor	Statistic	Annoyance of nonsmokers	Annoyance when smoking is prohibited	Recommend to quit smoking
Empathy	r	-0.025	.150	0.040
	p-value	0.747	.001	0.399
	N	165	452	453
Somatization	r	-0.125	-.119	-0.058
	p-value	0.110	.012	0.216
	N	165	452	453
Obsessive-compulsive	r	-0.080	0.020	0.045
	p-value	0.306	0.670	0.337
	N	165	452	453
Interpersonal sensitivity	r	-.227	-0.018	-0.047
	p-value	0.003	0.699	0.323
	N	165	452	453
Depression	r	-.285	0.008	0.020
	p-value	.001	0.860	0.671
	N	165	452	453
Anxiety	r	-.229	-0.044	0.022
	p-value	.003	0.349	0.642
	N	165	452	453
Hostility		-.272	-.116	-0.087
	p-value	.001	.013	0.065
	N	165	452	453
Phobic anxiety	r	0.025	-0.032	0.041
	p-value	0.746	0.496	0.385
	N	165	452	453
Paranoid ideation	r	-.218	-0.002	-0.084
	p-value	.005	0.969	0.073
	N	165	452	453
Psychoticism	r	-.268	-0.063	-0.026
	p-value	.001	0.179	0.582
	N	165	452	453
Global Severity Index	r	-.215	-0.040	-0.010
	p-value	.006	0.398	0.838
	N	165	452	453

**Table 19:** Independent samples t-test for psychological and empathy factors between smokers and nonsmokers in smoking-prohibited places. SD: standard deviation; df: degrees of freedom

Factor	Do not smoke in prohibited places (N=67), mean (SD)	Smoking in prohibited places (N=98), mean (SD)	t-test	df	P-value
Empathy	2.24 (0.80)	2.45 (0.74)	-1.709	163	0.089
Somatization	0.66 (0.68)	0.89 (0.66)	-2.162	163	.032
Obsessive-compulsive	0.88 (0.64)	1.02 (0.59)	-1.495	163	0.137
Interpersonal sensitivity	0.71 (0.60)	0.83 (0.67)	-1.239	163	0.217
Depression	0.88 (0.76)	0.87 (0.69)	0.133	163	0.894
Anxiety	0.62 (0.62)	0.78 (0.60)	-1.578	163	0.116
Hostility	0.78 (0.82)	1.31 (0.91)	-3.800	163	.001
Phobic anxiety	0.53 (0.62)	0.45 (0.58)	0.776	163	0.439
Paranoid ideation	0.76 (0.64)	1.14 (0.76)	-3.448	156.10	.001
Psychoticism	0.64 (0.55)	0.67 (0.55)	-0.366	163	0.714
Global Severity Index	0.73 (0.53)	0.86 (0.55)	-1.553	163	0.122

## Discussion

The current study aimed to identify the impact of smoking on psychology, empathy, and smoking behavior. The study also examined the effect of smokers’ psychological characteristics and empathy toward smoking behavior.

Smokers have a strong association between emotional intelligence and increased anxiety and depression [13]. Emotional intelligence demonstrates the ability of an individual to associate with those around them successfully and includes empathy and psychological state. Emotional intelligence is essential for assessing an individual’s mental state, explains aspects of human behavior, and focuses on the processing of situations faced by the individual, applying emotional and social context [13].

The first research question examined the association of smoking with psychological characteristics, empathy, and smoking behaviors. Smokers presented higher levels of hostility than nonsmokers, while participants who smoked more than 15 cigarettes per day indicated higher anxiety levels than those who smoked fewer than 15 cigarettes per day. According to Bernstein et al., aggressive responding is a risk factor for smoking [14]. Traditionally, studies on smoking describe behavior that can more appropriately be called “anxious mood” [15]. Gülsen et al. reported a positive correlation between high levels of nicotine addiction and high anxiety levels [16].

Smokers presented lower levels of empathy than nonsmokers when compared across the entire study population and within each sample category (e.g., employees, students, owners). All study participants stated that they seldom recommend that someone who smokes in a nonsmoking area quit smoking. Participants had low levels of somatization, obsessive-compulsive, interpersonal sensitivity, depression anxiety, hostility, phobic anxiety, paranoid ideation, and psychoticism. Empathy is a complex, multifaceted, dynamic concept that has been described in many ways and carries different meanings to different people. Empathy is a form of perception wherein observers genuinely feel the emotional states of others as their own [17]. A person’s level of empathy is associated with how they perceive another person and how they would attribute the other person’s behaviors as responsible for a difficult situation through the bias of their past experiences and future expectations [18]. Research has shown that social relationships are essential for physical and psychological well-being. Empathy helps regulate emotions and manage feelings, even in times of great stress, promoting supportive behaviors [19]. Romero et al. suggest that the lack of empathy is one of the reasons leading to smoking behavior [20].

Smokers presented low annoyance levels when someone else smoked in a smoking-prohibited area. They also had a low desire to ask a smoker to stop smoking in a nonsmoking area. Those who smoked more than 15 cigarettes had low levels of concern that nonsmokers in their presence are bothered by their smoking. Nonsmokers were more assertive against smoking exposure than fellow smokers, as reported previously [21].

We examined how smokers’ psychological characteristics and empathy affect their smoking behavior in enclosed public spaces and in front of nonsmokers. Smokers with higher interpersonal sensitivity, depression, anxiety, hostility, paranoid ideation, psychoticism, and generally stronger negative psychological symptoms did not think nearby nonsmokers are bothered by their smoking. Furthermore, smokers with higher somatization and hostility are less bothered when they are in a place where smoking is prohibited, and when someone else smokes. Smokers in nonsmoking areas had higher levels of somatization, hostility, and paranoid ideation. This finding agrees with those of Gülsen et al. who found that smokers show higher scores of somatization, anxiety, depression, hostility, and paranoia than nonsmokers and that the symptoms are more intense in people with high-grade nicotine addiction [16].

Smokers with high levels of empathy are more bothered when they are in a place where smoking is prohibited, and when someone else smokes. As Sayette et al. stated, smokers with empathy are usually in a “cold” state (i.e., trying to quit), while smokers with no empathy are in a “hot” state (i.e., in a high-craving state) [22]. When smokers are in a “hot” state, they are not bothered when they are in a place where smoking is prohibited, and when someone else smokes, and they might do the same.

According to studies that found that smokers experience psychopathological symptoms much more severely than nonsmokers, smoking might affect mental health [23]. Schizophrenia and diseases of the psychotic spectrum are of particular concern. Even though smoking is not explicitly associated with these symptoms, smokers with schizophrenia tend to have more severe symptoms than nonsmokers. A similar association is seen in depression and anxiety among smokers [24].

However, some myths link smoking to mental health, such as the flawed belief that smoking can help someone manage their mental health symptoms. Some mental healthcare professionals (such as psychologists or psychiatrists) tend to focus on other aspects of a person’s life in addressing their psychopathology while omitting their smoking habits; this might contribute to the belief that smoking helps relaxation and alleviates the symptoms of stress. In these cases, smoking cessation is not a priority [25].

Strengths and limitations

Primary research has the advantage of examining participants’ perceptions. However, a quantitative study is appropriate as empathy and psychological symptoms are measurable concepts and can be measured accurately using reliable and valid questionnaires [26]. This study assessed differences between groups (smokers and nonsmokers) and correlations within groups (correlation between variables) quantitatively as the assessments used statistical methods in numeric data [27]. Due to the inductive approach, quantitative study results can be generalized for the study population if the sample is representative [28]. Our results can be generalized for employees, owners, and medical and nonmedical students aged 18 to 30 years who are unmarried, have a bachelor’s degree, and are working full or part-time with high empathy and low psychosomatic symptoms.

Our study has significant limitations. Our sample of smokers was insufficient to use parametric tests that carry higher statistical power. In addition, this research was not experimental. Samples of smokers and nonsmokers were not similar regarding their demographic and occupational profiles. In particular, smokers were more likely to be males with high school education and vocational training. These differences may introduce biased results when evaluating the significance of comparing smokers and nonsmokers. Another limitation is that the part of the questionnaire regarding smoking behavior was primary and not examined for reliability and validity. Moreover, antismoking laws in Greece were not universally enforced and tended to be more enforced over time.

A future study with an adequate sample size for the statistical tests should be performed [29]. Sampling should be stratified to acquire a representative sample [28]. In a future study, the samples of smokers and nonsmokers should be demographically and occupationally similar to confirm a cause (i.e., smoking) and effect (i.e., consequences of smoking) relationship with fewer confounders than what was allowed in the current study design [30].

## Conclusions

This study explored the state of empathy among Greek smokers in public. Smokers presented higher levels of hostility than nonsmokers (regardless of occupation), and the level of anxiety was positively correlated with the number of cigarettes smoked per day. Smokers presented lower levels of annoyance when they were in a place where smoking is prohibited and when someone else smokes, as well as lower levels of effort to make a recommendation to someone who smokes in a nonsmoking area to quit. Smokers who smoked in prohibited places had higher levels of somatization, hostility, and paranoid ideation. Our results could assist the development of communication materials aimed at smokers to help them understand that others nearby do not enjoy their smoking practices, especially in an enclosed area. These findings could also facilitate feasible antismoking laws with an overall goal to reduce smoking in a population.
